# The Valence of Self-Generated (Status Updates) and Other-Generated (Wall-Posts) Information Determines Impression Formation on Facebook

**DOI:** 10.1371/journal.pone.0125064

**Published:** 2015-06-18

**Authors:** Harriet E. S. Rosenthal-Stott, Rea E. Dicks, Lois S. Fielding

**Affiliations:** Department of Psychology, Durham University, Durham, United Kingdom; Beihang University, CHINA

## Abstract

We examined whether self-generated (status updates) or other-generated (wall-posts) information on Facebook influenced the impression formed of the target individual. Along with examining reliance on particular types of information, we explored the valence (positive/ neutral/ negative) of the information, as reliance on self-generated or other-generated information may depend on whether self-presentation is perceived (i.e., presenting oneself positively / not negatively). Self-presentation may be perceived if the targets have positive/ neutral statuses, while negative statuses would indicate a lack of self-presentation. In line with previous research, participants should rely on other-generated information (wall-posts) to form an impression when participants are viewed to have self-presented (positive / neutral status updates), as this information could be viewed as unreliable. Forty participants rated nine Facebook profiles where statuses and wall-posts portrayed personality traits varying in valence. Each profile consisted of a neutral profile photo, three status updates (all positive, negative, or neutral) and three wall-posts (all positive, negative, or neutral). Materials were established in two pilots. Impression formation was measured as perceived social, task, and physical attractiveness of the target individual. Participants also ranked the profiles for likeability. Supporting our expectations, other-generated information (wall-posts) dominated impression formation for social attractiveness when self-generated information (status updates) was positive/ neutral. Task attractiveness was affected by information valence, regardless of source (self or other). Despite the inclusion of neutral photos, physical attractiveness was affected by self-generated information, with negative statuses lowering physical attractiveness. We suggest that these findings have implications for impression formation beyond the Facebook setting. The 557 traits analyzed in Pilot 1 are available as supporting information (S1 Dataset) and may be useful for other impression formation researchers.

## Introduction

When 17-year-old Ashleigh Hall befriended Peter Chapman on Facebook she thought she was chatting to a teenage boy, not to a 33-year-old convicted sex offender. When they met, he posed as his alter ego’s father, before suffocating her and dumping her body in a field [1]. With incidents like this reported in the media, and documentaries (e.g., *Catfish*) depicting instances where a person met online differs considerably from how they are in the real (offline) world, it is perhaps not surprising that people are cautious about relying on online identities when forming impressions [2]. However, it is not only online that people may attempt to manipulate others’ impressions of them. Self-presentation has been researched for over fifty-years [3], suggesting that a propensity to control others’ view of the self existed well before the birth of the internet. When forming an impression of an individual, people may be wary of this self-presentation motivation, and may therefore discount information given by the individual themselves. We explored this notion by examining whether information given by the individual (self-generated) was neglected in favor of information given by others, when forming impressions.

Two independent research fields have partially addressed this question. First, research into the *source effect* [4] established that ratings of a target for sociability/ competency were more in line with descriptions of the target written by a third-party, rather than descriptions written by the target themselves. This highlights that wariness of possible self-presentation can affect impression formation, such that the information given by the individual themselves is not trusted. Importantly, this source effect only occurred when descriptions were *positive*, due to a lack of perceived self-presentation bias for negative information. While this supports the idea that self-generated information is mistrusted, it does not address the question of whether self-generated information will be neglected when *both* self- and other-generated information are available.

Second, research specifically examining online impression formation has proposed that online presentation authenticates the actual, offline self [5–6]. Much like what is outlined above, this *warranting principle* suggests that other-generated information should be relied on for impression formation more than self-generated information, as self-generated information has a low warranting value and can be manipulated [7]. Some support has been found for this claim, as other-generated information (Facebook wall-posts–messages posted by friends) that consisted of either socially desirable or socially undesirable behavioral descriptions affected impression formation (social/ task attractiveness) [8]. However, the question of whether other-generated information is relied on above self-generated information was not addressed, as self-generated information was not included. Walther et al. [7] examined this question more directly, but with mixed results; for physical attractiveness, individuals were rated in line with other-generated (wall-posts), rather than self-generated (e.g., interests; activities) information (despite a neutral photo). However, this effect was not found for extraversion–which they suggested was because extraversion is not necessarily socially desirable (unlike physical attractiveness).

Research into the source effect and warranting principle highlight the propensity for self-generated information to involve self-presentation, suggesting that this information will be mistrusted when forming an impression. The warranting principle goes on to suggest that other-generated information should guide impression formation over and above self-generated information. However, there is a discrepancy between these two research fields, which involves the valence of the information. Brandt et al. [4] found that while others’ descriptions were a better predictor of impressions when the descriptions contained positive information, descriptions containing negative information produced negative impressions, regardless of the source. They suggested that this was because people do not tend to engage in negative self-presentation. As such, there is no need to be cautious about using self-generated negative information. In contrast, while Walther et al. [7] state that other-generated information will be relied on more than self-generated information when socially desirable (akin to positive) traits are involved, they do not theorize about whether self- or other-generated information (or both) will be relied on more when the individual is presenting themselves in an undesirable (akin to negative) manner.

Building on this prior research, we suggest that other-generated information will guide impression formation over self-generated information in cases where the self-generated information is positive/ neutral, as a self-presentation motive could exist. However, when the self-generated information is negative, no self-presentation motive exists; therefore, there is no need to disregard this information, making negative self-generated information useful for impression formation.

To explore this further, we examined impression formation in an online environment, focusing on the most popular social networking site (SNS)–Facebook [9]. Facebook is a useful setting to explore impression formation as while SNS profiles can reflect real-world (offline) personalities [10] and can yield similar impressions as real-world interactions [11], online impression formation may be affected by self-presentation processes [12]. Individuals rely on self-generated (provided by the profile owner, e.g., profile photo, personal information), other-generated (provided by friends, e.g., wall-posts), and system-generated (provided by Facebook, e.g., number of friends) information [13] to form an impression of the profile owner [14]. For self-generated cues, the profile owner can control what information is displayed in order to manage self-presentation [15]. Meanwhile, individuals have little control over other- and system-generated information, so these cues cannot be used to manage self-presentation.

### Current Research

We investigated the influence of self- and other-generated information on perceived attractiveness (social; physical; task) and ranked likeability of Facebook profile owners. Status updates (a self-generated cue that research so far has not examined), and wall-posts (one of the most salient cues for other-generated information [15]) referenced a selection of personality traits, which varied in valence (negative, positive, neutral) of social desirability. We focused on personality traits rather than positive / negative behavior [8], and included a variety of traits rather than a specific trait (e.g., sociability/ competency [4]; extraversion [7,13,16]). While all statuses/ wall-posts on a profile were of the same valence, valence between statuses and wall-posts either matched (e.g., positive statuses and positive wall-posts) or differed (e.g., positive statuses and negative wall-posts), creating nine profiles. Personality traits were established in Pilot 1.

McCroskey and McCain’s [17] interpersonal attractiveness measure (or its constituents: social; task; physical) has been used previously as a measure of online impression formation (e.g., [8], [13]). We were particularly interested in social attractiveness as this reflects the desire to form friendships–which is highly relevant to the SNS setting. However, task and physical attractiveness were included to assess whether effects would spillover to these domains. The profiles were also ranked for likeability.

Only information relating to the profile owner was provided, as information about friends (e.g., physical attractiveness [8]) can affect impressions. Only neutrally attractive profile pictures were included (established in Pilot 2), as physically attractive people may be viewed as more socially attractive [18]. We used female photos and a female only sample, as gender differences in impression formation have been noted [19] and there is a sexual double-standard for undesirable behavior (i.e., negative information)–with men perceived positively for engaging in socially undesirable behavior, and women perceived negatively [8].

We expected participants to mistrust positive/ neutral self-generated information, due to a possible self-presentation motivation, and instead rely on other-generated information (wall-posts). Therefore, if an individual describes themselves in a positive/ neutral manner, the extent to which they are perceived as socially attractive depends on the information given by their friends, with the most socially attractive profile being the one with positive wall-posts, followed by neutral wall-posts, followed by negative wall-posts. However, when an individual presents negative information, this cannot be seen as self-presentation, and thus there is no need to mistrust this information, and it can be relied on to form an accurate impression.

### Ethics Statement

Pilot 1, Pilot 2, and the main study were all approved by Durham University’s Department of Psychology Ethics Committee. All participants provided written consent.

## Pilot 1

To generate positive, negative, and neutral traits, Pilot 1 replicated Anderson (1968).

### Likeability/ Social Desirability

Seventy (36 female; 34 male) British undergraduates (*M*_*age*_ = 20.49; *SD*_*age*_ = 1.37) rated 557 traits [20] for how much they would like a person described by that word. The scale ranged from 0 (least favorable or desirable) to 6 (most favorable or desirable). Some traits were changed to British English based on spelling (e.g., honorable to honourable), or common usage (e.g., high-strung to highly-strung). A couple of additional alternatives were included (e.g., unconfident for nonconfident), increasing the original list from 555 to 557 traits. Trait order was randomized using a random list generator (www.random.org).

### Meaning

Thirty-two (16 female; 16 male) British undergraduates (*M*_*age*_ = 20.28; *SD*_*age*_ = 0.85) rated the 555 traits for how well they knew their meanings as descriptions of people. The scale ranged from 0 (I have almost no idea of the meaning of this word) to 4 (I have a very clear and definite understanding of the meaning of this word) [20].

### Results

Traits were excluded if likability varied across participants (*s* ^2^ > 1.50) or if meaning was not well understood (*M* < 3.70) [20]. Following exclusions, the 18 most desirable (positive) traits and 18 least desirable (negative) traits were selected. 18 neutral traits were also selected, however, due to experimenter error, the mid-point of 2.5 was used, rather than the mid-point of 3, making the neutral traits on the negative side of neutral.

The 555 traits, with likeability and meaning information, are included in the supporting information (S1 Dataset) and may be useful for other impression formation researchers.

## Pilot 2

Pilot 2 aimed to obtain photos of nine females who were statistically average for physical attractiveness.

### Method

Twenty female undergraduates (i.e., same gender as the main study participants) aged 18–21 (*M* = 20.05, *SD* = 0.95) viewed a PowerPoint presentation of 30 female photos taken from the 18–25 year old category of the photo rating website hotornot.com (for similar method see [8,15]). All photos were of women judged to be of university age, and were a mix of full-body and head-only shots to reflect variation in Facebook profile pictures. Photos were rated on a scale ranging from 1 (very unattractive) to 10 (very attractive).

### Results

One-sample *t*-tests generated exactly nine photos which did not significantly differ from the neutral score of 5.5, all *p*s>.05 (*p* = .079 to *p* = 1.00).

## Main Study: Method

### Participants & Design

Forty female undergraduates aged 18–22 (*M* = 19.73; *SD* = 1.11) participated in return for participation credit. All participants owned a Facebook profile, with usage ranging from 5 minutes a day to all-day (coded as 12 hours) (*M* = 2.02 hours; *SD* = 2.50). All participants used Facebook to keep in touch with friends; three participants also used Facebook to meet new people. Each participant rated all nine Facebook profiles.

### Procedure

Participants were given three minutes to read and examine print-offs of all nine profiles, in order to control for order effects. Participants then received the interpersonal attractiveness measure for each profile, which they completed in any order they wished, with all profiles still visible. The ranked likeability measure was then completed, followed by Facebook usage and demographics.

### Facebook profiles

Each profile included three statuses (matching in valence–i.e., all positive, all negative, or all neutral) written by the profile owner (self-generated) and three wall-posts (matching in valence) written by friends (other-generated). There were nine profile combinations: statuses (positive/ neutral/ negative) and wall-posts (positive/ neutral/ negative)

**Fig 1:**
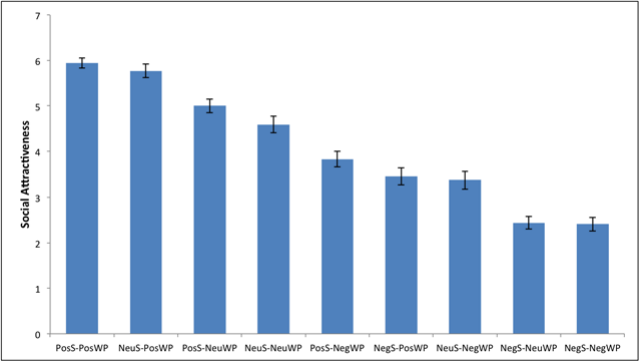
Social attractiveness ordered by profile mean. Error bars +/- 1 SE.

Profiles were generated from the information obtained from Pilot 1 and Pilot 2. Traits from Pilot 1 were embedded into a wall-post or status and randomly allocated to a profile. For example, “day spent volunteering at the hospital. can I get anymore kind-hearted? ♥” (kind-hearted: positive status); “cant [sic] believe you can be so dishonest. you could have just told me the truth in the first place….” (dishonest: negative wall-post). Intentional typographical and grammatical errors aimed to reflect Facebook’s relaxed writing style. Statuses and wall-posts were ordered randomly on each profile and each profile was randomly assigned a neutral photo from Pilot 2, along with a random British first (e.g., Charlotte, Emily) and last name (e.g., Williams, Cotton). Information about the friends (name; photo) was blacked out, so that only information about the profile owner was included in the profile. See Fig 1 and Table 1.

**Table 1:** Statuses and wall posts (trait in bold).

Profile	Statuses	Wall-posts
Positive statuses Positive wall-posts	hope he knows he’s not going to find anyone more **trustful** than me. / **Loyalty** means more than anything! I will always stick by my friends. / cant help being **cheerful** on a sunny day	Thank you so much for my present, you’re so **generous**! x / It means a lot to me that you were **truthful** about what happened on Friday. / I finally stopped laughing. . . thought about what you said and couldn’t stop again! You’re so **humorous**!
Neutral statuses Positive wall-posts	cant believe someone just text me from 3 floors up telling me I am so **noisy** they could hear me on the phone outside! haha / why am I so **compulsively** obsessed with desperate housewives?????? / why am I so **unlucky**. . . second time in three days a bird has pooed on me!: (: (	I’m glad you took the joke in a **good humoured** way! haha x / Cannot wait to see you’re **happy** beaming face: D ♥ / Don’t worry about you’re meeting tomorrow, I’m sure they’ll love you, you’re such a **likeable** person! xxxxx
Positive statuses Neutral wall-posts	Personally I find myself quite **amusing**! / I am always **honest**. . . it’s definitely the best policy. / drama drama drama could my life get any more **interesting** right now	you sometimes remind me of a mouse because your so **timid** lol x / I never thought I’d see the day you would **conform** and apply for the typical graduate jobs / Let your hair down! You don’t need to be **reserved** all the time! x
Neutral statuses Neutral wall-posts	Lost my keys AGAIN. . . god I’m so **forgetful**! x / My middle name must be **clumsy** / good **gossip** with the girls	As if you believed laidback luke was djing at my party haha you’re so **gullible** x / your so **untidy**, please please please clean up your crap! x / LOL at your **bluntness x**
Positive statuses Negative wall-posts	Just been told I was one of the **friendliest** people they have ever met:) / Come down to the fair tonight, I will be there to give everyone a **warm** welcome!!! / day spent volunteering at the hospital. can I get anymore **kind-hearted**? ♥	can’t believe you can be so **dishonest**. . . You could have just told me the truth in the first place. . . / being that critical was just **unkind**: (/ there was no need to be like that, you are supposed to give constructive criticism, not just be **ultra-critical.**
Negative statuses Positive wall-posts	Is it bad to enjoy being **cruel**?!?!?! / don’t even care I’m **selfish**, you’ve got to look after number 1:) / being nice gets you nowhere, welcome to the new **unfriendly** me!	hahaha As if you did that! You’re so **comical**!!!! x / I knew you were **trustworthy**, thank you so much! ♥ ♥ ♥ / Thank you sooo much, you’re too **kind**! xxxxxxx
Neutral statuses Negative wall-posts	ewww cannot actually believe adeles making me watch embarrassing bodies! I’m so **squeamish**: (/ Eurghhhhhh I don’t know what to do with my lifeeee! Im so **undecided**! / so **nervous** about the show tonight!!!	Becky I did think you were being a bit **mean** last Friday. . . / God how **malicious** were you last Friday, there was just no need for it. / I honestly think you are the most **spiteful** person I have ever met!!!!
Negative statuses Neutral wall-posts	you know I am **untrustworthy** so why tell me?!? / Yes I’m **hostile**. . . I don’t like you. GET OVER IT! / parents just ranted at me about being **deceitful**. like I care!	come on your turn to decide what we’re doing. stop being so **indifferent** x / you were so **showy** dancing in the street last night xx / thanks for bringing the work in, I knew I could **depend** on you! xx
Negative statuses Negative wall-posts	im such a good **liar**, my parents will never know! / **offensive** is better than defensive / So what if I’m **insulting**, I tell the truth!	You’re just pure **abusive.** / speaking to someone like that is just **heartless** Pippa! / stop being so **dull**… come out tonight!

### Measures

#### Interpersonal attraction

The interpersonal attraction scale [17] measured social (e.g., *I think she could be a friend of mine)*, task (e.g., *If I wanted to get things done*, *I could probably depend on her*), and physical (e.g., *I think she is quite pretty*) attractiveness. Degree of agreement with each of the 15 items ranged from 1 (totally disagree) to 7 (totally agree). Following reverse coding, a higher mean indicated higher attractiveness.

#### Ranked likability

Participants ranked the profiles for likability by placing a number next to the profile owners’ names, indicating the most likable profile owner (1) to the least likable profile owner (9).

#### Facebook usage and demographics

Participants were asked if they owned a Facebook profile, how often they used Facebook, and their reasons for using it [21]. Demographic information was also collected.

## Main Study: Results

### Social Attractiveness

A status x wall-post repeated measures ANOVA revealed a significant main effect for status, F(2,78) = 139.82, *p* < .001, η^2^_p_ = .782. Bonferroni adjusted comparisons established that positive status profiles (*M* = 4.92) were more socially attractive than neutral (*M* = 4.58), *p* = .041, and negative (*M* = 2.77), *p* < .001, status profiles. Neutral status profiles were more socially attractive than negative status profiles, *p* < .001. The main effect for wall-post was also significant, F(2,78) = 98.97, *p* < .001, η^2^_p_ = .717. Positive wall-post profiles (*M* = 5.05) were more socially attractive than neutral (*M* = 4.01), *p* < .001, and negative (*M* = 3.20), *p* < .001, wall-post profiles. Neutral wall-post profiles were more socially attractive than negative wall-post profiles, *p* < .001. The significant status x wall-post interaction, F(4,156) = 8.76, *p* < .001, η^2^_p_ = .183, was explored with Bonferroni adjusted comparisons within each status and wall-post valence (see Table 2 for *M*s and *SD*s).

**Table 2:** Interpersonal Attraction.

Social Attractiveness				Physical Attractiveness				Task Attractiveness			
Statuses	Wall-Posts	M	SD	Statuses	Wall-Posts	M	SD	Statuses	Wall-Posts	M	SD
Positive	Positive	5.94	0.72	Neutral	Positive	4.66	0.97	Positive	Positive	5.41	0.69
Neutral	Positive	5.77	0.97	Positive	Neutral	4.57	0.90	Neutral	Positive	4.99	0.94
Positive	Neutral	5.01	0.93	Neutral	Negative	4.48	1.10	Positive	Neutral	4.72	1.15
Neutral	Neutral	4.59	1.15	Positive	Positive	4.46	0.98	Positive	Negative	4.18	0.92
Positive	Negative	3.83	1.18	Neutral	Neutral	4.45	1.02	Negative	Positive	4.17	1.18
Negative	Positive	3.45	1.17	Positive	Negative	4.12	1.22	Negative	Neutral	3.45	1.22
Neutral	Negative	3.38	1.21	Negative	Positive	3.97	1.16	Neutral	Negative	3.05	0.93
Negative	Neutral	2.44	0.87	Negative	Negative	3.86	1.08	Neutral	Neutral	2.96	1.02
Negative	Negative	2.41	0.94	Negative	Neutral	3.68	1.47	Negative	Negative	2.92	0.87

Profiles ordered by mean social attractiveness, mean physical attractiveness, and mean task attractiveness.

#### Within each status valence

When profiles had positive statuses, the positive wall-post profile was rated more socially attractive than the neutral, *p* < .001, and negative, *p* < .001 wall-post profiles. The neutral and negative wall-post profiles were also significantly different, *p* < .001. The same pattern emerged when statuses were neutral; the positive wall-post profile was rated more socially attractive than the neutral, *p* < .001, and negative, *p* < .001, wall-post profiles. The neutral and negative wall-post profiles were also significantly different, *p* < .001. This suggests that the participants are using the wall-posts to form impressions when the statuses are positive or neutral.

When the statuses were negative, the positive wall-posts profile was rated more socially attractive than the neutral, *p* < .001, and negative, *p* < .001, wall-post profiles, but there was no significant difference between the neutral and negative wall-post profiles, *p* = 1.00. These findings suggest that wall-post valence was taken into account when forming an impression, when profiles contained positive/ neutral statuses. However, when the self-generated information was negative, this was relied on to form a (negative) impression, which was boosted only when positive other-generated information was present.

#### Within each wall-post valence

When wall-posts were positive, there was no significant difference between the positive and neutral status profiles, *p* = .759. However, the negative status profile was significantly different from both the positive, *p* < .001, and neutral, *p* < .001, status profiles. The same pattern occurred for the neutral wall-post profiles; there was no significant difference between the positive and neutral status profiles, *p* = .295, but the negative status profile was significantly different from both the positive, *p* < .001, and neutral, *p* < .001, status profiles. This pattern was again reflected for the negative wall-post profiles; there was no significant difference between the positive and neutral status profiles, *p* = .219, but the negative status profile was significantly different from both the positive, *p* < .001, and neutral, *p* < .001, status profiles. With the same pattern for all three wall-post valences, there is clear support for the idea that other-generated information dominates impression formation, except in cases where self-generated information is negative.

### Task Attractiveness

A status x wall-post repeated measures ANOVA revealed a significant main effect for status, F(2,78) = 38.35, *p* < .001, η^2^_p_ = .496. Bonferroni adjusted comparisons established that positive (*M* = 4.77) status profiles were higher in task attractiveness than neutral (*M* = 3.66), *p* < .001, and negative (*M* = 3.51), *p* < .001, status profiles. However, there was no significant difference between the neutral and negative status profiles, *p* = 1.00. The main effect for wall-post was also significant, F(2,78) = 77.00, *p* < .001, η^2^_p_ = .664. Positive (*M* = 4.85) wall-post profiles were higher in task attractiveness than neutral (*M* = 3.71), *p* < .001, and negative (*M* = 3.38), *p* < .001, wall-post profiles. Neutral wall-post profiles were higher in task attractiveness than negative wall-post profiles, *p* = .003. The status x wall-post interaction was significant, F(4,156) = 7.77, *p* < .001, η^2^_p_ = .166, and explored further by Bonferroni adjusted comparisons for each status and wall-post valence.

#### Within each status valence

When statuses were positive, the positive wall-post profile was higher in task attractiveness than the neutral, *p* = .004, and negative, *p* < .001, wall-post profiles. The difference between the neutral and negative wall-post profiles was marginally non-significant, *p* = .057. When statuses were neutral, a similar pattern emerged. The positive wall-post profile was higher in task attractiveness than the neutral, *p* < .001, and negative, *p* < .001, wall-post profiles. There was no significant difference between the neutral and negative wall-post profiles, *p* = 1.00. When statuses were negative, the positive wall-post profile was higher in task attractiveness than the neutral, *p* = .018, and negative, *p* < .001, wall-post profiles. There was also a significant difference between the neutral and negative wall-post profiles, *p* = .018.

#### Within each wall-post valence

When wall-posts were positive, the positive wall-post profile was higher in task attractiveness than the neutral, *p* = .011, and negative, *p* < .001, status profiles. The difference between the neutral and negative status profiles was also significant, *p* = .006. For the neutral wall-post profiles, the positive status profile was higher in task attractiveness than the neutral, *p* < .001, and negative, *p* < .001, status profiles. There was no significant difference between the neutral and negative status profiles, *p* = .232. The same pattern was reflected for the negative wall-post profiles; the positive status profile was higher in task attractiveness than the neutral, *p* < .001, and negative, *p* < .001, status profiles, and there was no significant difference between the neutral and negative status profiles, *p* = 1.00.

These findings do not support other-generated information as dominating impression formation over self-generated information for task attractiveness. Instead, it appears that positive information, whether statuses or wall-posts, results in higher task attractiveness, while negative information (and to a certain extent, neutral information), results in lower task attractiveness.

### Physical Attractiveness

Despite Pilot 2 selecting neutrally attractive photos, a status x wall-post repeated measures ANOVA revealed a significant main effect for status, F(2, 78) = 14.64, *p* < .001, η^2^_p_ = .273. Bonferroni adjusted Pairwise comparisons revealed no significant difference between the positive (*M* = 4.38) and neutral (*M* = 4.53) status profiles, *p* = .877. However, profiles with negative statuses (*M* = 3.84) were significantly less physically attractive than both the positive, *p* = .002, and neutral *p* < .001 status profiles. Both the main effect for wall-post (*M*_*positive*_ = 4.36; *M*_*neutral*_ = 4.23; *M*_*negative*_ = 4.15), F(2, 78) = 1.91, *p* = .155, η^2^_p_ = .047, and the status x wall-post interaction, F(4,156) = 1.17, *p* = .328, η^2^_p_ = .029, were non-significant (see Table 2 for *M*s and *SD*s). Negative self-generated information appears to result in lower perceived physical attractiveness.

### Ranked Likability

Friedman Test analysis revealed a significant difference between the nine profiles for ranked likability, χ^2^(8) = 229.16, *p* < .001. With the exception of the ‘negative-statuses-and-positive-wall-posts’ profile and the ‘positive-statuses-and-negative-wall-posts’ profile (which swapped positions) ranked order matched profiles ordered by social attractiveness. See Table 3.

**Table 3:** Ranked Likeability.

Statuses	Wall-Posts	*M* Likability Ranking	*SD*	Median	Min	Max
Positive	Positive	1.75	0.98	1	1	4
Neutral	Positive	1.95	0.96	2	1	5
Positive	Neutral	3.45	1.38	3	1	7
Neutral	Neutral	4.10	1.55	4	1	8
Negative	Positive	5.58	1.68	5.5	1	9
Positive	Negative	6.05	1.87	6	2	9
Neutral	Negative	6.38	1.68	6	3	9
Negative	Neutral	7.60	1.15	8	5	9
Negative	Negative	8.18	0.93	8	6	9

Means and SD for the ranked likability of each profile (1 = most likeable; 9 = least likeable), including the median rank, and minimum to maximum rank.

## Discussion

Results for social attractiveness supported our hypotheses that: (1) the extent to which an individual is perceived as socially attractive depends on the information given by their friends, if they describe themselves in a positive/ neutral manner; (2) when an individual presents negative information, this will be relied on to form an impression. For profiles with positive/ neutral statuses, social attractiveness was highest for positive wall-post profiles, second-highest for neutral wall-post profiles, and lowest for negative wall-post profiles. This suggests that, in line with our theorizing, participants were relying on other-generated information to form impressions. However, negative statuses did guide impression formation, resulting in lower social attractiveness (boosted when positive other-generated information was present). Thus, other-generated information appears to dominate impression formation, except in cases where self-generated information is negative.

This finding agrees with expectations based on the source effect [4] and warranting principle [5], by adding support to the idea that self-generated information will be mistrusted (due to a possible self-presentation motivation). However, this result broadens these research fields by: (1) examining self- and other-generated information concurrently; and (2) exploring the valence of the information. In doing so, we have bridged the gap between these two fields. We can now state that other-generated information guides impression formation (social attractiveness) over self-generated information in cases where the self-generated information is positive or neutral. However, when the self-generated information is negative, it can be perceived as useful for impression formation. We assume that the difference between positive/ neutral and negative self-generated information is due to a difference in perceived self-presentation motivation.

Although social attractiveness was our primary concern, task and physical attractiveness were included to establish whether the effect would spillover into these other impression formation domains. Task attractiveness did not clearly follow the pattern for social attractiveness. Instead of other-generated information dominating impression formation, level of task attractiveness had more to do with valence. Positive information, whether self- or other-generated, resulted in higher task attractiveness than both neutral and negative information. This suggests self-presentation motivations may not be of importance when examining task attractiveness. This may be due to the nature of task attractiveness, which is a measure of how easy or worthwhile working with the individual would be [17]. In this context, it would probably be easier to work with somebody with a positive personality, than a negative (or neutral) personality, regardless of whether or not this reflects their true personality. In contrast, when looking for a friendship (i.e., social attractiveness) we have a desire to form an accurate impression of that person’s personality. This finding reflects previous research where those with positive other-generated information were rated higher in task attractiveness than those with negative other-generated information [8], although we extend this to establish the same effect for self-generated information (not previously examined).

Results for physical attraction were also noteworthy. While Pilot 2 carefully selected neutrally attractive photos, a significant difference based on self-generated information still emerged. Specifically, negative status profiles were less physically attractive than the positive and neutral status profiles, suggesting that negative self-generated information lowers perceived physical attractiveness. While previous research has established the ‘what is beautiful is good’ [18] and the ‘what is good is beautiful’ [22] concepts, our findings suggest ‘what is bad is ugly’. Also, the lack of effects for other-generated information contrasts a previous finding that other-generated information is relied on more than self-generated information when making physical attractiveness judgments [7]. However, the self- and other-generated information used by Walther et al. [7] focused on physical attractiveness, while our information related to personality traits, which may explain the difference between the two studies. Likewise, positive other-generated information (attractive friends/ socially desirable wall-posts) has previously been found to increase perceived physical attractiveness for female profile owners, compared to negative other-generated information [8]. As self-generated information was not included in that study, it may be that other-generated information is used to judge physical attractiveness only in the absence of self-generated information.

## Conclusions

While we use Facebook as the setting for our research, we believe that the implications of our findings extend to other impression formation domains, such as meeting potential new friends on the first day of school/ university, and chatting (online or offline) to a potential romantic partner. Our findings suggest that if you provide positive (or neutral) information about yourself, the extent to which you will be viewed as socially attractive depends on what others say about you. Positive information provided by others leads to a good impression (socially attractive), providing that you do not provide negative information. In essence, the key to forming a good impression is to avoid mentioning any negative information about yourself, and to get good feedback from others (preferable positive, but never negative). In other words, always come armed with a good reference letter from friends/ lovers, and hide that deep, dark personality flaw.

## Supporting Information

S1 Dataset Pilot 1 data. (XLS)

S2 Dataset Pilot 2 data. (XLS)

S3 Dataset Main study likeability data. (XLS)

S4 Dataset Main study ranking data. (XLS)
